# Molecular Basis, Diagnostic Challenges and Therapeutic Approaches of Alport Syndrome: A Primer for Clinicians

**DOI:** 10.3390/ijms222011063

**Published:** 2021-10-14

**Authors:** Raquel Martínez-Pulleiro, María García-Murias, Manuel Fidalgo-Díaz, Miguel Ángel García-González

**Affiliations:** 1 Grupo de Xenética e Bioloxía do Desenvolvemento das Enfermidades Renais, Laboratorio de Nefroloxía (No. 11), Instituto de Investigación Sanitaria de Santiago (IDIS), Complexo Hospitalario de Santiago de Compostela (CHUS), 15706 Santiago de Compostela, Spain; raquel.martinez.pulleiro0@usc.es (R.M.-P.); Maria.Garcia.Murias@sergas.es (M.G.-M.); 2 Grupo de Medicina Xenómica (GMX), 15706 Santiago de Compostela, Spain; 3 Departamento de Nefrología, Complexo Hospitalario Universitario de Santiago (CHUS), 15706 Santiago de Compostela, Spain; Manuel.Fidalgo.Diaz@sergas.es; 4 Fundación Pública Galega de Medicina Xenómica-SERGAS, Complexo Hospitalario de Santiago de Compostela (CHUS), 15706 Santiago de Compostela, Spain

**Keywords:** Alport syndrome, hereditary kidney disease, chronic kidney disease, collagen, *COL4A*

## Abstract

Alport syndrome is a genetic and hereditary disease, caused by mutations in the type IV collagen genes *COL4A3*, *COL4A4* and *COL4A5*, that affects the glomerular basement membrane of the kidney. It is a rare disease with an underestimated prevalence. Genetic analysis of population cohorts has revealed that it is the second most common inherited kidney disease after polycystic kidney disease. Renal involvement is the main manifestation, although it may have associated extrarenal manifestations such as hearing loss or ocular problems. The degree of expression of the disease changes according to the gene affected and other factors, known or yet to be known. The pathophysiology is not yet fully understood, although some receptors, pathways or molecules are known to be linked to the disease. There is also no specific treatment for Alport syndrome; the most commonly used are renin–angiotensin–aldosterone system inhibitors. In recent years, diagnosis has come a long way, thanks to advances in DNA sequencing technologies such as next-generation sequencing (NGS). Further research at the genetic and molecular levels in the future will complete the partial vision of the pathophysiological mechanism that we have, and will allow us to better understand what is happening and how to solve it.

## 1. Introduction

**Figure 1:**
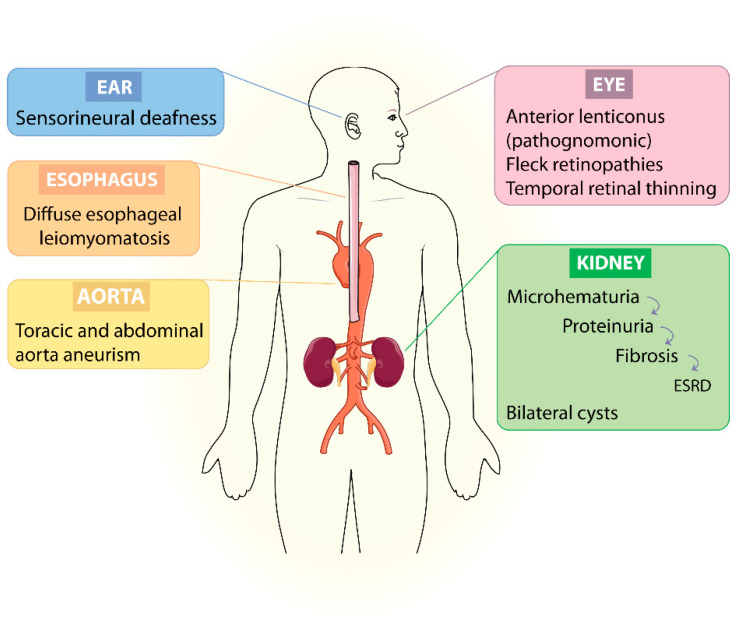
Main defining phenotypic characteristics of Alport syndrome.

Alport syndrome (AS) is a rare monogenic hereditary disorder caused by mutations in any of the type IV collagen genes *COL4A3*, *COL4A4* (2q36.3 both) and *COL4A5* (Xq22.3). They encode collagen chains α3, α4 and α5, present in the glomerular basement membrane (GBM), among other basement membranes of the organism. AS is defined by a hematuric nephritis that can be accompanied by sensorineural deafness, ocular defects and other less common extrarenal manifestations [1] (Figure 1).

### 1.1. Clinical Manifestations

α3, α4 and α5 collagen chains are located in the basement membranes of the glomerulus, Bowman’s capsule, distal tubules in the kidney, cochlea, retina, cornea, lens capsule, skin and smooth muscle cells [2,3]. Therefore, their deficiency or modification can affect to a lesser or greater extent the function of the kidney, ear, eyes, skin and muscle.

Renal damage is the phenotype with the worst consequences for health. The first manifestation is microhematuria, which may occur intermittently. Hematuria may progress to proteinuria, loss of renal function and end-stage renal disease (ESRD). Sometimes, after renal transplantation, IgG deposits are found in the graft, although post-transplant anti-GBM disease develops very rarely [4,5]. Recently, mutations in type IV collagen genes were proposed to cause bilateral cysts after a whole-exome sequencing (WES) investigation. According to the authors, studies with larger cohorts are required to verify this relationship. If confirmed, the phenotypic spectrum of AS could be broadened and explain cases of polycystic kidney disease in which no mutation is detected [6,7].

Bilateral sensorineural hearing loss (BSHL) is a common feature of AS, and whenever it is expressed, it is accompanied by renal symptoms. BSHL causes a decrease in sensitivity to medium and high frequencies. BSHL associated with AS appears and progresses over time. About 18% of children (2 to 18 years old) develop hearing loss, while in adulthood it occurs in 70% of cases. It can be considered as a prognostic factor for progressive kidney disease, i.e., the earlier the onset of BSHL, the greater the probability of developing ESRD [8,9].

The most common ocular manifestations are lenticonus and fleck retinopathy. The first one is pathognomonic of AS and consists of a bulging of the lens capsule that makes focusing difficult. Its diagnosis is usually made after the onset of renal failure and in conjunction with hearing loss [8]. Lenticonus is correctable. Central and peripheral fleck retinopathies are both retinal abnormalities related to early-onset renal failure. Temporal retinal thinning is also very common in AS when compared with other kidney diseases [10]. Fortunately, these abnormalities do not cause vision loss or may be correctable. At the ocular level, there are other features that are also linked to AS but rarely appear [11]. 

**Figure 2:**
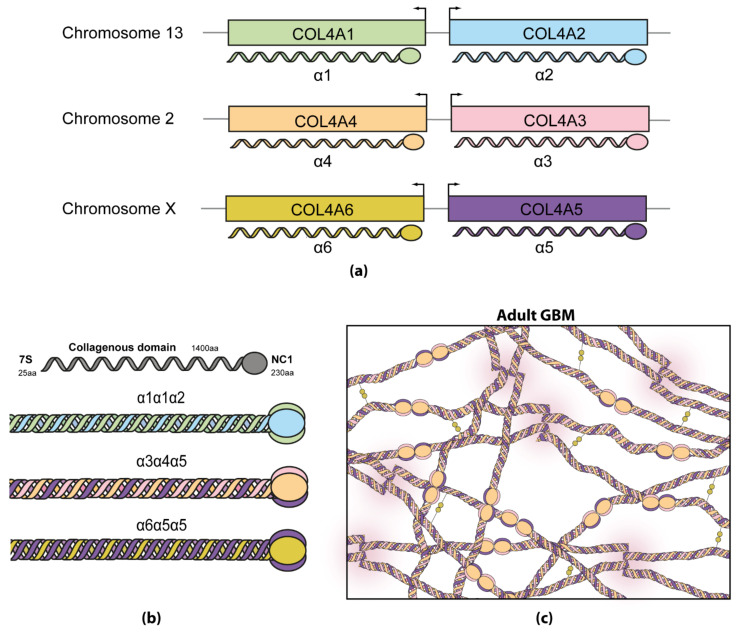
(**a**) Type IV collagen genes (COL4A1–COL4A6) are located in three different chromosomes pairwise, that encode the corresponding α-chains (α1–α6). (**b**) α-chains can be combined among each other in three different ways, forming triple helices (trimers). (**c**) In adults, collagen α3α4α5 trimers associate by C and N termini, creating a crosslinked network reinforced by disulfide bonds (gold circles).

Diffuse leiomyomatosis (DL) is a benign smooth muscle tumor condition that affects mainly and firstly the esophagus, although it can also affect the gastrointestinal or female genital tract. In the vast majority of cases, DL-AS (MIM#308940) is caused by deletions involving 5’ ends of *COL4A5* and *COL4A6* genes and the common promoter regions in between. It is important to know that these two genes are found together head to head on chromosome X and that they share the same promoter (Figure 2a). However, a couple of cases have also been published in which deletions in *COL4A6* or in the promoter region are not essential for the development of DL [2,12,13].

In very few cases, there have been adverse cardiovascular events such as aneurysms or dissections [14,15].

### 1.2. Clinical Presentation

AS can present a wide phenotypic variability, ranging from isolated hematuria to kidney failure, depending on the type of inheritance, type of mutation and mutation position. AS can be transmitted in three inheritance patterns: X-linked, autosomal recessive and autosomal dominant.

X-linked inheritance (XLAS) is the main form of AS (MIM#301050), representing about 80% of cases. Males with this condition have hemizygous mutations in the *COL4A5* gene and they are severely affected, with a 60% probability of starting ESRD before 30 years old, and 90% by age 40 years [16]. Hearing loss occurs in 90% of men with XLAS before the age of 40 [17] and about 30% of males with XLAS suffer from ocular defects [18]. Women who carry heterozygous mutations in *COL4A5* may have a mild phenotype with hematuria or one as severe as males. To explain this variability, one of the factors that has been proposed is the random inactivation of chromosome X in females, demonstrating the role of epigenetics on AS expression and progression. It is estimated that women with XLAS have a possibility of 12% of suffering kidney failure by the age of 40 years, and 15–30% by age 60 years [19]. Hearing loss is a frequent event in women with XLAS (28%), but usually occurs after the age of 30–40 years. The risk of having ocular defects is around 15% [17]. 

Autosomal-recessive AS (ARAS) caused by mutations in homozygosis or compound heterozygosis in *COL4A3* or *COL4A4* genes (MIM#203780) has a similar phenotype to that of X-linked inheritance in males [20]. 

Heterozygous mutations in *COL4A3* (MIM#104200) or *COL4A4* cause autosomal-dominant AS (ADAS), with a wide spectrum of phenotypes [21]. Most patients develop a mild phenotype but some of them (29%) progress and can reach ESRD later in life [22,23]. Extrarenal manifestations are unusual [24]. The variability can be so wide that even members of the same family, with the same variant, can express the disease differently [25]. Although cases due to dominant inheritance were thought to be the fewest in the past, a next-generation sequencing (NGS) study revealed that they account for 31% of cases [23].

Some cases of AS can also be explained by a digenic inheritance among collagen genes, which has a better prognosis than XLAS/ARAS [22,26,27].

It has become clear, especially in recent years, that the inheritance of AS is more complex and more difficult to explain than the typical Mendelian inheritance. Type of mutation also influences the severity of the disease. Males with XLAS are the ones that have a stronger correlation between genotype and phenotype. Population studies reveal missense mutations as the ones with the best prognosis, with a later onset of ESRD (37 years on average), while large rearrangements and mutations that lead to stop codons have the worst prognosis, developing ESRD in the early 20s [28]. The development of hearing and ocular changes is associated with the type of the mutation in the same way as ESRD. Not only the type of mutation, but also the position of the mutation play a role in the development of the disease. Mutations positioned at the 5’ end of *COL4A5* gene are related to an early age of onset of ESRD and also with the appearance and severity of hearing and ocular changes [16,18]. Collagen gene mutations can also interact with mutations in other genes, creating a complex phenotype [29,30]. Other genetic, epigenetic and environmental factors that have not yet been discovered are probably influencing phenotypic variability. 

**Figure 3:**
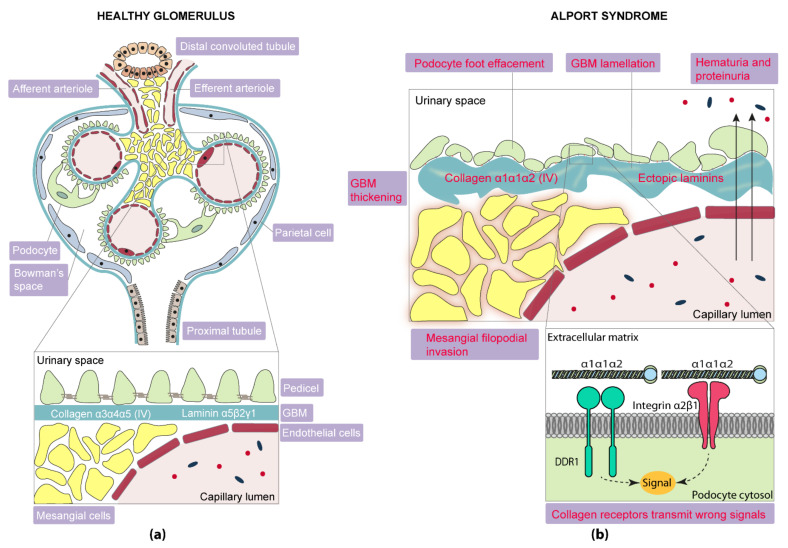
Graphic representation of the renal glomerulus and detail of the glomerular filtration barrier. (**a**) Healthy glomerulus presents podocyte foot processes with slit diaphragms. The mature GBM is composed of collagen α3α4α5 and laminin α5β2γ1. Albumin does not filtrate pathologically into the urinary space. (**b**) In Alport glomerulus, podocyte foot effacement disrupts the podocyte structure and slit diaphragms disappear. Immature forms of collagen and laminins are expressed in the GBM as a compensatory mechanism. Albumin is lost pathologically due to increased permeability.

Histological analysis of glomeruli with AS shows segmental thinning and thickening of the GBM, podocyte foot process effacement and mesangial proliferation. GBM can also undergo lamellation, typical of AS [31] (Figure 3). An injury pattern of focal segmental glomeruloesclerosis (FSGS) can also be present by light microscopy. 

### 1.3. Prevalence

The prevalence of classic AS is estimated to be around 1:5000–10,000 live births, and it is considered a rare disease [32]. These data could change if the information is synthesized, and we begin to classify based on the genetic cause. In the past, mild cases of hematuria were usually classified as benign familial hematuria (BFH) or thin basement membrane nephropathy (TBMN), but both diseases have their origin in mutations in autosomal *COL4A* genes, usually in heterozygosity. If all three diagnoses, AS, BFH and TBMN, were considered as one entity based on genetics (collagen nephropathy), and if we take into account undiagnosed and misdiagnosed cases, the prevalence is estimated to be much higher [33]. Population-based cohort genetic analysis showed that AS is the second most common inherited kidney disease, behind autosomal-dominant polycystic kidney disease (ADPKD) [34].

## 2. Molecular Basis of the Disease

### 2.1. In Vitro and In Vivo Modeling of Alport Syndrome

Animal models have been the main tool to investigate AS pathophysiology, as they represent a complex biological system similar to that of humans, on a small scale and with a short lifespan. For the investigation of AS, murine models are the most commonly used. There are mouse lines with mutations in the *Col4a3* or *Col4a4* genes for the study of ARAS, and in *Col4a5* for the study of XLAS. The strains that have been used so far are briefly shown in Table 1. The models mimic human AS, presenting progressive glomerulonephritis with microhematuria and proteinuria. Histologically, thickening and thinning of the GBM and fibrosis can be observed. Genetic background plays a role in disease progression, e.g., C57BL/6 Col4a3 mice progress more slowly and reach ESRD later than 129 Alport mice. The longer survival can be explained, in part, by the ectopic deposition of α5α5α6 collagen in C57BL/6 [35,36]. The model with the 129 background has been the most chosen by the scientific community for the study of AS. Recently, a genetically diverse XLAS mouse model has been developed from random crosses between known founder strains. The goal is to simulate the phenotypic variability of AS that we often observe. This new approach makes it possible to find disease-modifying genes [37]. Mouse animal models were also used to derive primary cultures of mesangial cells and podocytes [38,39]. In the past, dogs with spontaneous mutations in type IV collagen genes were used to investigate the pathophysiology of Alport syndrome [35].

### 2.2. Collagen in Alport Syndrome

The glomerular basement membrane (GBM) is a specialized extracellular matrix (ECM) synthetized by the fenestrated endothelium, which covers the membrane on the capillary side, and the podocytes that cover it on the urinary space side. The three layers together form the glomerular filtration barrier (GFB). The major components of the GBM are laminin, heparan sulphate proteoglycan, nidogen and type IV collagen, the latter being the most abundant [50]. There are six type IV collagen genes (*COL4A1*–*COL4A6*) that encode six different collagen chains, which assemble to form three combinations of sterically compatible heterotrimers: α1α1α2, α3α4α5 and α5α5α6 (Figure 2a,b). All chains share a common structure, composed by a short N-terminal 7S domain (25 amino acids), a long collagenous domain (approximately 1400 amino acids) and a C-terminal non collagenous domain (NC1) (approximately 230 amino acids) (Figure 2b). The collagenous domain has the typical repetitive amino acid sequence Gly-X-Y, where X is normally proline and Y is normally 4-hidroxyproline, with a fundamental role in the assembly of the heterotrimer [51]. Collagen chains are assembled in the endoplasmic reticulum and secreted to the extracellular space [52]. Assembly starts from the C-terminal NC1 end [53] and depends on the formation of disulfide bonds between cysteine residues [52,54] and the stabilization by sulfilimine bonds [50,55] and chloride ions [53,56].

The α1α1α2 heterotrimer is characteristic during embryogenesis in all basement membranes of the body. During development, basement membranes change their composition, replacing the α1α1α2 heterotrimer with α3α4α5 in the GBM, cochlea, eyes, testes and lungs, and with the α5α5α6 heterotrimer in skin, smooth muscle, Bowman’s capsule and distal tubules in the kidney [2,3]. In the GBM, α3α4α5 heterotrimers are synthesized and secreted solely by podocytes [3]. The network built by the crosslinking of the α3α4α5 heterotrimers is more stable than that formed by α1α1α2, since it better withstands the stress of filtration and is less susceptible to proteolysis by matrix metalloproteinases [51]. In AS, a mutation in any of the three genes *COL4A3*, *COL4A4* or *COL4A5* will lead to the absence or dysfunctional formation of α3α4α5 heterotrimers, and the persistence of α1α1α2 heterotrimers [3].

### 2.3. Other Glomerular Alterations in Alport Syndrome

The collagen defect triggers the appearance of several modifications at the molecular level, leading to the appearance of physiological alterations. Some of the most characteristic ones are described below.

#### 2.3.1. Laminins

Laminins are organized in heterotrimers and, in GBM, switch from immature isoforms, α1β1γ1 (laminin 111) and α5β1γ1 (laminin 511), to a mature isoform, α5β2γ1 (laminin 521), and both were produced by endothelial cells and podocytes [57,58] (Figure 3a). The reason why this substitution happens remains unknown; however, it may be necessary to resist the hydrostatic pressure exerted by the blood that circulates through the glomerular capillaries. Aberrant deposits of laminin α2 were found in XLAS mouse, dog and human as part of laminin α2β1γ1 (laminin 211) and α2β2γ1 (221), and in humans only as part of the laminin 221 (Figure 3b). Laminin α2 correlates with the activation of focal adhesion kinase (FAK) in podocytes. Laminin 111 re-expression was revealed in Col4a3 Alport mice, in both endothelial cells and podocytes. Nonetheless, this re-expression does not happen in humans with XLAS, in whom laminin α1 is not found in the GBM when immunostained [59,60]. Laminin 111 and 211 tend to accumulate in areas of irregular thickening [61]. 

#### 2.3.2. Receptors

##### Integrins

Integrins are transmembrane receptors that communicate with each other and with the extracellular environment. Integrins have been shown to play a fundamental role in the development of the renal glomerulus. In Alport models, integrins have been studied and their role in the pathogenesis of the disease has been elucidated. A Col4a3 Alport mouse (129 Sv/J) shows an overexpression of vimentin and integrin α3 in podocytes and of integrin α1 in mesangial cells [62]. Integrin α1β1 regulates matrix metalloproteinases via p38 mitogen-activated protein kinase in mesangial cells, increasing the expression of MMP2, MMP9 and MMP14 in α1 integrin-null mice and in α1 integrin-null Alport mice. MMP9 levels are also elevated in Col4a3 Alport mice (129 Sv/J) via the ERK pathway [63]. Integrin α1β1/Rac1 mediates the mesangial cell process invasion of the capillary loop due to an increased migration capacity in Alport mice. Laminin α2 also has a role in this invasion [61]. Integrin αvβ6 is upregulated in Col4a3 Alport mouse kidneys (129 Sv/J), especially in cortical tubular epithelial cells. This molecule has a role in the AS fibrosis process as it is able to activate TGF-β. In an Col4a3 Alport mouse model, tubulointerstitial fibrosis was dramatically inhibited after immunoblocking or knock-out of αvβ6 [64].

##### Collagen Receptors

Impaired type IV collagen could transmit wrong signals or stop transmitting the correct signals. The α1α1α2 chains that remain in AS are located close enough to the podocytes to interact with their membrane collagen receptors [65].

Podocyte collagen receptors, discoidin domain receptor 1 (DDR1) and integrin α2β1, are upregulated in AS and both have been described to play a relevant role in renal fibrosis, highlighting the importance of cell–matrix communication (Figure 3b). Comparing animal models of AS that express *Ddr1* at different doses (*Ddr1*^+/+^ *Col4a3*^−/−^; *Ddr1*^+/−^ *Col43*^−/−^; *Ddr1*^−/−^ *Col4a3*^−/−^), it was observed that the DKO (double knockout) model maintains podocyte structure, and develops less fibrosis at the glomerular and interstitial level. DKO improves survival and kidney function [66]. The mechanism of action that links the receptor to fibrosis has not yet been elucidated. Recently, DDR1 activation has been linked with a lipotoxic effect in podocytes mediated by CD36 [39]. The intervention of any of these pathways associated with this tyrosine kinase receptor represents an opportunity for the treatment of AS. By using a parallel DNA-encoded library screening, an inhibitor of DDR1 (2.45) autophosphorylation was found that prevents its activation by collagen in renal epithelial cells. The improvement in renal function after the use of 2.45 in a Col4a3 Alport mice model (129 Sv/J) is comparable to that obtained by the knocking out of the gene encoding DDR1 [67].

Loss of integrin α2β1 (*Itga2*^−/−^ *Col4a3*^−/−^ mice) reduces interstitial fibrosis and glomerulosclerosis, improves the GBM ultrastructure and delays ESRD when compared to the *Itga2*^+/+^ *Col4a3*^−/−^ animal model [68]. 

The role of DDR2 (discoidin domain receptor 2), highly similar to DDR1, was investigated in an X-linked AS mouse (B6). DDR2, despite having high expression levels in AS, does not have a clear implication in the pathogenesis of AS [69].

##### CC Chemokine Receptor 2

As previously mentioned, abnormal expression of matrix metalloproteinases has been detected in AS glomeruli. MMP12 has a more than 40-fold higher expression in the AS glomerulus than in a normal renal glomerulus. The same occurs in the glomeruli of humans and dogs. There is evidence that this upregulation is promoted by the activation of the CC chemokine receptor 2 (CCR2) in Col4a3 Alport mice (129 Sv/J) podocytes. The inhibition of MMP12 showed a restoration of the GBM ultrastructure, which recovered a uniform thickness, and podocytes recovered their foot processes and slit diaphragms. This pathway, in macrophages, is related to acute and chronic inflammatory responses [70].

#### 2.3.3. Mesangial Filopodial Invasion

Biomechanical strain induces the expression of endothelin-1 (ET-1) in endothelial cells from the glomeruli. These peptides induce the activation of endothelin receptor type A (ET_A_R) in mesangial cells, which triggers the mesangial filopodial invasion of glomerular capillaries via Rac1/CDC42 (Figure 3b). Mesangial filopodia deposit mesangial proteins in the GBM, such as laminin α2, able to activate focal adhesion kinase (FAK) in podocytes. This signaling cascade ends up activating a pro-inflammatory response [38]. Inhibiting FAK activation with TAE226 decreases the glomerular expression of matrix metalloproteinase 9 (MMP9), MMP10 and MMP12, partially restoring kidney function in a Col4a3 Alport mouse model (129 Sv/J) [71].

#### 2.3.4. Permeability

The Col4a3 Alport mouse (129 Sv/J) was also used to test the permeability of Alport GBM compared to WT GBM. After ferritin injection in both mice, by confocal microscopy, more ferritin was seen throughout the Alport GBM, especially in areas of thickening and podocyte foot effacement. In addition, the accumulation of laminin α1 and α5 were detected in thickening areas [72]. All these changes gradually cause the glomerular structure to deteriorate (Figure 3b). 

#### 2.3.5. Podocyte Detachment

Population studies demonstrated that AS patients are born with the same number of podocytes as healthy people; however, they suffer an accelerated podocyte detachment, with an 11-fold change increase over the years, which can be measured in the urine [73]. 

#### 2.3.6. Fibrosis

Glomerular damage triggers a pro-inflammatory response to try to recover normal kidney function. As the system cannot correct the genetic deficiency, ECM begins to be deposited in an abnormal way, turning into fibrotic tissue that loses its functional capacity. Fibrosis is a common precursor to chronic kidney disease (CKD). This process involves multiple molecules, such as proinflammatory cytokines, chemokines, growth factors, signaling and transcription factors or macrophages. In AS, fibrosis develops at the tubulointerstitial and glomerular levels [74]. Fibrosis is irreversible, so the treatment of AS, like that of other fibrotic diseases, is aimed at preventing or slowing tissue fibrosis.

## 3. Diagnosis

### 3.1. Clinical Diagnosis

The importance of diagnosis lies in being able to provide treatment, and obtain the maximum benefit from the treatment. The sooner you receive treatment, the better the results will be in terms of disease progression and entry into ESRD. Clinical suspicion of AS can arise based on different data, such as finding hematuria or proteinuria in urinalysis, a family history of renal disease, the expression of ocular/hearing defects or a kidney biopsy showing pathological signs. Nevertheless, these symptoms are not unique to AS:

- Hematuria and proteinuria could be caused by many diseases that affect the glomerulus and the blood filtration process.
- Familial information could be useful, although not in all cases. In ARAS, cases can skip generations, and it is estimated that 10–15% of the cases of men with XLAS are due to *de novo* mutations [8].
- Extrarenal manifestations are hardly ever present, only in the most severe cases.
- The most common histological sign of AS is FSGS, which can be caused by other genetic or non-genetic diseases. However, it was recently described that mutations in *COL4A* genes explain 38% of cases of familial FSGS and 3% of sporadic FSGS [75]. Skin biopsy could also be useful in cases of XLAS, although it is currently an obsolete technique [54]. Histological techniques have been used for decades for the diagnosis of AS and innovations are ongoing. A new immunostaining technique is able to differentiate AS with incidental IgA deposits from IgA nephropathy [76].

By themselves, none of these parameters give a clear diagnosis of AS, but in combination they can. 

### 3.2. Genetic Diagnosis

Additionally, as the causal genes are known, a genetic approach can be used to diagnose AS and thus learn more about the origin of the disease. If there is suspicion of typical AS, the confirmation can be achieved simply by sequencing a gene panel that includes *COL4A3*, *COL4A4* and *COL4A5* genes. Otherwise, when there are doubts about the diagnosis, it is recommendable to use a wide screening strategy by using a comprehensive gene panel, whole-exome sequencing (WES) or whole-genome sequencing (WGS). If family history does not clarify the inheritance pattern, genetic testing would help to reveal it. The genetic variants found should be interpreted according to current knowledge and expert recommendations [31]. A large number of mutations are known to cause AS; some of them are mutations unique to particular families and others are recurrent. (http://www.hgmd.cf.ac.uk/ac/ (accessed on 6 October 2021)). When sequencing does not find any variant that explains the phenotype, it is advisable to search for CNVs (copy number variants) by alternative techniques such as multiplex ligation-dependent probe amplification (MLPA). Deletions and insertions explain approximately 10% of AS cases. Variants are more difficult to identify if they are of deep intronic splicing or are mosaically found. All other rearrangements have to be confirmed by a second method (haplotype analysis, comparative genomic hybridization array or customized MLPA analysis) [77].

Nowadays, direct sequencing of *COL4A* genes by the Sanger method is rarely used to diagnose, even if the suspicion of Alport is very clear. NGS has evolved so much in recent years that with little effort and for a reasonable price, a great deal of information can be obtained. The results make it possible to know the causative mutation, located in *COL4A* genes, and other possible variants that may be modifying the phenotype. Another advantage of gene panels is that they allow carrying out a differential diagnosis in a single step. In case the patient suffers from a genetic disease with clinical features similar to AS, the panel will reveal what this other disease is [3]. The use of Sanger sequencing, at the diagnostic level, has been restricted to carry out segregation studies.

### 3.3. Prognosis

Apart from continuing to improve the diagnosis, currently, it is intended to identify prognostic biomarkers of the disease. The samples used to look for biomarkers are plasma, serum and urine. Finding a good biomarker in urine is preferable, as the sample collection is non-invasive for the patient. Studies analyzing potential biomarkers are complex, as a large number of patients and controls are needed, as well as the monitoring of disease progression for a subsequent correlation between biomarker trend and progression. A biomarker of progression in children could be uEGF/Cr (urinary epidermal growth factor normalized by urine creatinine). A lower expression level of uEGF/Cr in AS patients than in healthy individuals predicts a higher risk of progression, although glomerular filtration rate values remain preserved [78]. Nowadays, the only way to predict disease progression to ESRD is the appearance of risk factors (increasing proteinuria or hearing loss) or renal insults (nephrotoxic medication, kidney donor). 

## 4. Treatment

AS manifests and progresses differently in each patient. The currently used treatments aim to slow the progression of the disease, thus achieving a delay in kidney failure and prolonging lifespan. Nonetheless, the majority of patients still require renal replacement therapy [79]. Renin–angiotensin–aldosterone system (RAAS) inhibition by angiotensin-converting enzyme (ACE) inhibitors and angiotensin receptor blockade (ARB) is the mainly chosen strategy for treating AS, due to its proven antihypertensive, antiproteinuric and nephroprotective effect [80]. In fact, ACE inhibitors are the first-line therapy and AT1-receptor antagonists are used as the alternative to ACE inhibitors if they cause any kind of inconvenience in the patient. An increase in RAAS axis activity is related to the progression of CKD through hemodynamic (increased pressure in the efferent arteriole) and non-hemodynamic processes (increased cytokine production). Blockade of the RAAS system prevents these pathological processes and makes the kidney condition improve and remain in good condition for a longer time. The combination of RAAS inhibitors with second therapies has been shown to increase the efficiency of the treatment, although the risk of side effects is also higher [81].

Recently, a population with XLAS of 430 male patients was studied and disclosed a significant difference in the median age of onset of ESRD between treated and untreated patients (50 years vs. 28 years, respectively). Correlating these data with the genotype, patients with truncating mutations were also seen to delay their ESRD onset when treated (16 years vs. 28 years) [82]. In the same way, heterozygous XLAS carriers were proven to have reduced lifespan compared with healthy controls, and treatment with RAAS inhibitors significantly delayed the onset of ESRD [83]. A retrospective study of 101 ARAS patients was evaluated, noting also a nephroprotective effect of RAAS therapy. Patients with impaired kidney function experienced a delay of CKD stage G5 by an average of 11 years thanks to treatment (CKD G5 24 years old untreated vs. 35 years old treated) [84]. No treated patients with microhematuria progressed to kidney failure. These findings agree with a previous study, in which a comparison of untreated and treated relatives at different disease stages (impaired renal function, proteinuria, hematuria/microalbuminuria) exposed that the benefit of treatment is greater the earlier it is started. Early-treated patients may need renal replacement therapy, with a lapse of 13 years in comparison to later or non-treated siblings [85]. It is fundamental to start treatment before the onset of proteinuria, because by itself it is a factor of kidney damage [86]. 

Ramipril, an ACE inhibitor, demonstrated efficacy and safety in a pediatric AS population in the EARLY PROTECT ALPORT clinical trial (NCT01485978) (Table 2), as it had previously in animal models [87,88]. All this evidence led experts in the field to revisit the diagnosis and management of AS in children, adolescents and young adults. In 2020, they published an updated guideline underlining an early start of the treatment regime with RAAS inhibitors and so on, highlighting the relevance of early diagnosis [89]. In general terms, we can consider that there are different stages along the evolution of the disease. In the first years of life, renal function is maintained (normal GFR and without albuminuria) and if we wanted to see in young patients a sign derived from the disease, it would be a thinning of the GBM and perhaps microhematuria. In school years, the GMB begins to thicken until proteinuria and fibrosis appear in adolescence [90]. Treating the pediatric population is very important because of the great qualitative leap in terms of possible benefits. Males with XLAS and patients with ARAS are recommended to start treatment with RAAS inhibitors at the time of diagnosis. However, females with XLAS and patients with ADAS should start it when the first signs of the disease appear (microalbuminuria) [89].

### 4.1. Clinical Trials for Alport Syndrome

Apart from RAAS inhibitors, some other candidate drugs have been or are being tested to treat AS in clinical trials (Table 2).

- Bardoxolone is an anti-inflammatory agent that acts by activating the transcription factor Nrf2 (erythroid 2-related factor 2) and inhibiting the NF-κB (kappa-light-chain-enhancer of activated B cells) pathway [91]. The safety and efficacy of bardoxolone was evaluated in the CARDINAL clinical trial (NCT03019185). A total of 187 adult and pediatric participants at various stages of the disease, with and without previous ACEi/ARB treatment, were enrolled in this study. Long-term safety is now being evaluated in a phase 3 EAGLE clinical trial (NCT03749447) that includes 480 participants.
- In a Col4a3 Alport mouse model (129 Sv/J), paricalcitol demonstrated renal protective and antifibrotic effects. Paricalcitol was assessed along with an ACE inhibitor and the results show a synergistic effect capable of delaying ESRD onset [92]. This drug is being tested in an observational clinical trial (NCT02378805).
- The HERA clinical trial (NCT02855268) is now recruiting for a phase 2 interventional study of lademirsen (previously known as RG-012), an inhibitor of miR-21. In vivo experiments have shown how the silencing of this miRNA reduces the inflammation and fibrosis of AS [93].
- Atrasentan is a selective endothelin A receptor antagonist that reduces albuminuria without causing fluid retention, as other members of its family do [94]. It has been assessed in the SONAR clinical trial (NCT01858532) in diabetic patients and the final results show that the risk of kidney events decreases, protecting renal function [95]. Currently, the AFFINITY clinical trial (NCT04573920) is recruiting for testing atrasentan in proteinuric glomerular diseases, including AS.
- Spirinolactone is an aldosterone antagonist that could help treat AS in those cases in which ACE inhibitors lose effectivity. In a Col4a3 Alport animal model (129 Sv/J), a concomitant treatment of ACE and spironolactone reduced proteinuria levels and fibrosis. However, the premature death of some mice could be a side effect of the treatment [96]. Adverse effects of this combination have been shown also in humans [97]; therefore, it must be administered under strict supervision. Spirinolactone effects in humans are being tested in an observational clinical trial (NCT02378805).
- HMG-CoA reductase inhibitors or statins are well known for their action in regulating cholesterol levels. Nonetheless, anti-inflammatory and anti-fibrotic effects have been associated with them, which are clearly of interest in AS. An example is cerivastatin, which is able to reduce proteinuria and fibrosis, prolonging the lifespan of the Alport mice model (129 Sv/J) [98]. The results of the use of statins to treat AS are being evaluated in an observational study (NCT02378805).
- Sparsentan is a dual-acting drug, angiotensin II type 1 (AT1) receptor and endothelin A receptor (ETAR) blocker. The clinical trial EPPIK (NCT05003986) is still recruiting to test sparsentan for the treatment of various proteinuric glomerular diseases, including AS in a pediatric population. Sparsentan has given good results treating FSGS in a phase 2 DUET clinical trial (NCT01613118) [99] and is currently in phase 3 (NCT03493685) [100].

In order to obtain the best possible results, the Alport Syndrome Foundation (ASF) published a guide with recommendations for the approach to clinical trials [101]. At present, the treatments that are reaching the clinical trial phases are drugs, although there are new therapy ideas in preclinical trials.

### 4.2. Pre-Clinical Trials for Alport Syndrome

Some other drugs are being proved in animal models and, moreover, new methods of administering drugs are being sought to achieve better drug effectiveness and to avoid unwanted effects on the body. In 2021, an in vivo study with metformin in the Col4a5 mutant Alport model (B6) confirmed the improvement in the state of inflammation and fibrosis after the administration of the drug [102]. Olmesartan prevents tubulointerstitial fibrosis by downregulating TGFβ in Col4a3 Alport mice (129X1/SvJ) [103]. Such antifibrotic action is maintained when olmesartan is targeted specifically to the kidney using hydrophobically modified glycol chitosan (HGC) nanomicelles, while avoiding side effects such as hypotension [104].

## 5. Conclusions and Future Perspectives

AS is a chronic disease that makes life difficult for those who suffer from it. XLAS and ARAS often lead to renal failure at an early age, causing patients to have to undergo renal replacement therapy, with all that this entails.

It is very important to take advantage of the resources available. At present, it is essential to make a genetic diagnosis as soon as possible and to prescribe the necessary treatment, trying to achieve the greatest benefit for the patient, prolonging his state of well-being as long as possible. Right now, sequencing techniques are available, efficient and affordable. In terms of treatment, it is capable of slowing the progression of the disease, and the earlier it is started the better. Starting the treatment at the correct moment should be the goal for as many patients as possible.

Molecular research of the disease has slowed down in recent years. The most fundamental discoveries were made years ago, and since then the greatest advances have been in the fields of pharmacology and genetics. Of course, the latter is fundamental and meaningful, but it is essential to know what happens between the beginning (genetic origin) and the end (symptoms and their treatment). Deciphering the unknowns of the pathophysiology of AS is always going to be positive; the more we know, the better we will be able to manage the disease. In a few years, perhaps, when we talk about the treatment of AS, we will not only refer to drugs, as is the case today, but also to other alternative therapies, with action at the gene, RNA or protein level, which are on the right track [51]. The more we advance in disease research and the more we learn about the disease, the more innovative ideas will emerge and the closer we will come to curing the disease.

## Figures and Tables

**Table 1:** Murine animal models of human Alport syndrome.

Strain	Gene	Genetics	References
129-Col4a3^tm1Dec^/J	*COL4A3*	*Col4a3^tm1Dec^/Col4a3^tm1Dec^*	[40]
129X1/SvJ-Col4a3^tm1Dec^	*COL4A3*	*Col4a3^tm1Dec^/Col4a3^tm1Dec^*	[41]
129S1/Sv * 129X1/SvJ	*COL4A3*	*Col4a3^tm1Jhm^/Col4a3^tm1Jhm^*	[42]
129S1/Sv * 129S6/SvEvTac * 129X1/SvJ	*COL4A3*	*Col4a3^tm1Jhm^/Col4a3^tm1Jhm^* *Mmp9^tm1Tvu^/Mmp9^tm1Tvu^*	[43]
129X1/SvJ * C57BL/6	*COL4A3*	*Col4a3^tm1Dec^/Col4a3^tm1Dec^*	[40]
129S1.NON(NZO)-Col4a4^bwk^/PgnJ	*COL4A4*	*Col4a4^bwk^/Col4a4^bwk^*	[44]
C57BL/6J-Col4a4^m1Btlr^	*COL4A4*	*Col4a4^m1Btlr^/Col4a4^m1Btlr^*	[45]
D2.NON(NZO)-Col4a4^bwk^/GrsrJ	*COL4A4*	*Col4a4^bwk^/Col4a4^bwk^*	[44]
C3H/HeH * C57BL/6J	*COLA4A*	*Col4a4^m1H^/Col4a4^m1H^*	[36]
NON;NZO-Col4a4bwk/J	*COL4A4*	*Col4a4^bwk^/Col4a4^bwk^*	[44]
B6.Cg-Col4a5^tm1Yseg^	*COL4A5*	*Col4a5^tm1Yseg^/Col4a5^+^*	[46,47]
B6.Cg-Col4a5^tm1Yseg^	*COL4A5*	*Col4a5^tm1Yseg^/Y*	[46,47]
C57BL/6J-Col4a5^em1Keha^	*COL4A5*	*Col4a5^em1Keha^/Y*	[48]

* Model name according to MGI (Mouse Genome Informatics)© [49].

**Table 2:** Clinical trials that have tested the efficacy of drugs for the treatment of Alport syndrome and their current status.

Identifier	Study	Status	Interventions	Characteristics	Population	Sponsor
NCT01485978	Efficacy and Safety Study to Delay Renal Failure in Children with Alport Syndrome	Completed	Drug: ramipril Drug: placebo to ramipril	Phase 3	From 24 months to 18 years	Institut fuer anwe dungsorientierte Forschung und klinische Studien GmbH University Medical Center Goettingen German Federal Ministry of Education and Research
NCT03019185	A Phase 2/3 Trial of the Efficacy and Safety of Bardoxolone Methyl in Patients With Alport Syndrome -CARDINAL	Completed	Drug: placebo oral capsule Drug: bardoxolone methyl	Phase 2 Phase 3	From 12 years to 60 years	Reata Pharmaceuticals, Inc.
NCT03749447	An Extended Access Program for Bardoxolone Methyl in Patients with CKD (EAGLE)	Recruiting	Drug: bardoxolone methyl	Phase 3	Of 12 years and older	Reata Pharmaceuticals, Inc.
NCT02378805	European Alport Therapy Registry European Initiative Towards Delaying Renal Failure in Alport Syndrome	Recruiting	Drug: ACE inhibitor Drug: AT1 inhibitor Drug: HMG coenzyme inhibitor (statin) Drug: spironolactone Drug: paricalcitol	Observational	Child, adult, older adult	University Hospital Goettingen Society for Pediatric Nephrology (Germany) Deutsche Gesellschaft für Nephrologie Alport Selbsthilfe e.V. Association pour l’Information et la Recherche sur les Maladies Rénales Génétiques (AIRG) KfH Foundation Preventive Medicine
NCT02855268	Study of Lademirsen (SAR339375) in Patients with Alport Syndrome	Recruiting	Drug: lademirsen (SAR339375) Drug: placebo	Phase 2	From 18 years to 55 years	Genzyme, a Sanofi Company Sanofi
NCT04573920	Atrasentan in Patients with Proteinuric Glomerular Diseases	Recruiting	Drug: atrasentan	Phase 2	Of 18 years and older	Chinook Therapeutics U.S., Inc. Chinook Therapeutics, Inc.
NCT05003986	Study of Sparsentan Treatment in Pediatrics with Proteinuric Glomerular Diseases	Recruiting	Drug: sparsentan	Phase 2	From 1 year to 17 years	Travere Therapeutics, Inc.

Status according to https://clinicaltrials.gov/, accessed on 9 September 2021.

## References

[1] Stokman M.F., Renkema K.Y., Giles R.H., Schaefer F., Knoers N.V.A.M., Van Eerde A.M. (2016). The expanding phenotypic spectra of kidney diseases: Insights from genetic studies. Nat. Rev. Nephrol..

[2] Sá M.J.N., Fieremans N., De Brouwer A.P.M., Sousa R., Costa F.T., Brito M.J., Carvalho F., Rodrigues M., de Sousa F.T., Felgueiras J. (2013). Deletion of the 5′exons of COL4A6 is not needed for the development of diffuse leiomyomatosis in patients with Alport syndrome. J. Med. Genet..

[3] Kruegel J., Rubel D., Gross O. (2013). Alport syndrome—Insights from basic and clinical research. Nat. Rev. Nephrol..

[4] Byrne M.C., Budisavljevic M.N., Fan Z., Self S.E., Ploth D.W. (2002). Renal transplant in patients with Alport’s syndrome. Am. J. Kidney Dis..

[5] Mallett A., Tang W., Clayton P.A., Stevenson S., McDonald S.P., Hawley C.M., Badve S.V., Boudville N., Brown F.G., Campbell S.B. (2014). End-stage kidney disease due to Alport syndrome: Outcomes in 296 consecutive Australia and New Zealand dialysis and transplant registry cases. Nephrol. Dial. Transplant..

[6] Gulati A., Sevillano A.M., Praga M., Gutierrez E., Alba I., Dahl N.K., Besse W., Choi J., Somlo S. (2020). Collagen IV Gene Mutations in Adults With Bilateral Renal Cysts and CKD. Kidney Int. Rep..

[7] Sevillano A.M., Gutierrez E., Morales E., Hernandez E., Molina M., Gonzalez E., Praga M. (2014). Multiple kidney cysts in thin basement membrane disease with proteinuria and kidney function impairment. Clin. Kidney J..

[8] Kashtan C.E. (2001). Collagen IV-Related Nephropathies (Alport Syndrome and Thin Basement Membrane Nephropathy). GeneReviews™.

[9] Boeckhaus J., Strenzke N., Storz C., Gross O. (2020). Characterization of sensorineural hearing loss in children with alport syndrome. Life.

[10] Chen Y., Colville D., Ierino F., Symons A., Savige J. (2018). Temporal retinal thinning and the diagnosis of Alport syndrome and Thin basement membrane nephropathy. Ophthalmic Genet..

[11] Savige J., Sheth S., Leys A., Nicholson A., Mack H.G., Colville D. (2015). Ocular features in Alport syndrome: Pathogenesis and clinical significance. Clin. J. Am. Soc. Nephrol..

[12] Zhou J., Mochizuki T., Smeets H., Antignac C., Laurila P., De Paepe A., Tryggvason K., Reeders S.T. (1993). Deletion of the paired α5 (IV) and α6 (1V) collagen genes in inherited smooth muscle tumors. Science.

[13] Nozu K., Minamikawa S., Yamada S., Oka M., Yanagita M., Morisada N., Fujinaga S., Nagano C., Gotoh Y., Takahashi E. (2017). Characterization of contiguous gene deletions in COL4A6 and COL4A5 in Alport syndrome-diffuse leiomyomatosis. J. Hum. Genet..

[14] Kashtan C.E., Segal Y., Flinter F., Makanjuola D., Gan J.-S., Watnick T. (2010). Aortic abnormalities in males with Alport syndrome. Nephrol. Dial. Transplant..

[15] Patel J., Abt P., Cheng K., Aurigemma G., Rosenthal L. (2020). Type A Dissection in a Patient with Alport Syndrome. Circ. Cardiovasc. Imaging.

[16] Jais J.P., Knebelmann B., Giatras I., de Marchi M., Rizzoni G., Renieri A., Weber M., Gross O., Netzer K.-O., Flinter F. (2000). X-linked Alport Syndrome: Natural History in 195 Families and Genotype_Phenotype Correlations in males. J. Am. Soc. Nephrol..

[17] Jais J.P., Knebelmann B., Giatras I., De Marchi M., Rizzoni G., Renieri A., Weber M., Gross O., Netzer K.O., Flinter F. (2003). X-linked Alport syndrome: Natural history and genotype-phenotype correlations in girls and women belonging to 195 families: A “European Community Alport Syndrome Concerted Action” study. J. Am. Soc. Nephrol..

[18] Bekheirnia M.R., Reed B., Gregory M.C., McFann K., Shamshirsaz A.A., Masoumi A., Schrier R.W. (2010). Genotype-phenotype correlation in X-linked Alport syndrome. J. Am. Soc. Nephrol..

[19] Savige J., Colville D., Rheault M., Gear S., Lennon R., Lagas S., Finlay M., Flinter F. (2016). Alport syndrome in women and girls. Clin. J. Am. Soc. Nephrol..

[20] Storey H., Savige J., Sivakumar V., Abbs S., Flinter F.A. (2013). COL4A3/COL4A4 mutations and features in individuals with autosomal recessive alport syndrome. J. Am. Soc. Nephrol..

[21] Pescucci C., Mari F., Longo I., Vogiatzi P., Caselli R., Scala E., Abaterusso C., Gusmano R., Seri M., Miglietti N. (2004). Autosomal-dominant Alport syndrome: Natural history of a disease due to COL4A3 or COL4A4 gene. Kidney Int..

[22] Fallerini C., Baldassarri M., Trevisson E., Morbidoni V., La Manna A., Lazzarin R., Pasini A., Barbano G., Pinciaroli A.R., Garosi G. (2017). Alport syndrome: Impact of digenic inheritance in patients management. Clin. Genet..

[23] Fallerini C., Dosa L., Tita R., Prete D.D., Feriozzi S., Gai G., Clementi M., La Manna A., Miglietti N., Mancini R. (2014). Unbiased next generation sequencing analysis confirms the existence of autosomal dominant Alport syndrome in a relevant fraction of cases. Clin. Genet..

[24] Furlano M., Martínez V., Pybus M., Arce Y., Crespi J., Venegas M.d.P., Bullich G., Domingo A., Ayasreh N., Benito S. (2021). Clinical and Genetic Features of Autosomal Dominant Alport Syndrome: A Case Series. Am. J. Kidney Dis..

[25] Altun I., Saygılı S., Canpolat N., Özlük Y., Hürdoğan Ö., Yeşil G., Çalışkan S., Sever L. (2021). Strong mesangial IgA staining—Does it always refer to IgA nephropathy in a patient with proteinuria and hematuria? Answers. Pediatr. Nephrol..

[26] Mencarelli M.A., Heidet L., Storey H., Van Geel M., Knebelmann B., Fallerini C., Miglietti N., Antonucci M.F., Cetta F., Sayer J.A. (2015). Evidence of digenic inheritance in alport syndrome. J. Med. Genet..

[27] Daga S., Fallerini C., Furini S., Pecoraro C., Scolari F., Ariani F., Bruttini M., Mencarelli M.A., Mari F., Renieri A. (2019). Non-collagen genes role in digenic alport syndrome. BMC Nephrol..

[28] Barua M., Paterson A.D. (2021). Population-based studies reveal an additive role of type IV collagen variants in hematuria and albuminuria. Pediatr. Nephrol..

[29] Frese J., Kettwig M., Zappel H., Hofer J., Gröne H.J., Nagel M., Sunder-Plassmann G., Kain R., Neuweiler J., Gross O. (2019). Kidney injury by variants in the COL4A5 gene aggravated by polymorphisms in slit diaphragm genes causes focal segmental glomerulosclerosis. Int. J. Mol. Sci..

[30] Voskarides K., Papagregoriou G., Hadjipanagi D., Petrou I., Savva I., Elia A., Athanasiou Y., Pastelli A., Kkolou M., Hadjigavriel M. (2018). COL4A5 and LAMA5 variants co-inherited in familial hematuria: Digenic inheritance or genetic modifier effect?. BMC Nephrol..

[31] Savige J., Ariani F., Mari F., Bruttini M., Renieri A., Gross O., Deltas C., Flinter F., Ding J., Gale D.P. (2019). Expert consensus guidelines for the genetic diagnosis of Alport syndrome. Pediatr. Nephrol..

[32] Odiatis C., Savva I., Pieri M., Ioannou P., Petrou P., Papagregoriou G., Antoniadou K., Makrides N., Stefanou C., Ljubanović D.G. (2021). A glycine substitution in the collagenous domain of Col4a3 in mice recapitulates late onset Alport syndrome. Matrix Biol. Plus.

[33] Warady B.A., Agarwal R., Bangalore S., Chapman A., Levin A., Stenvinkel P., Toto R.D., Chertow G.M. (2020). Alport Syndrome Classification and Management. Kidney Med..

[34] Groopman E.E., Marasa M., Cameron-Christie S., Petrovski S., Aggarwal V.S., Milo-Rasouly H., Li Y., Zhang J., Nestor J., Krithivasan P. (2019). Diagnostic Utility of Exome Sequencing for Kidney Disease. N. Engl. J. Med..

[35] Cosgrove D., Kalluri R., Miner J.H., Segal Y., Borza D.B. (2007). Choosing a mouse model to study the molecular pathobiology of Alport glomerulonephritis. Kidney Int..

[36] Falcone S., Wisby L., Nicol T., Blease A., Starbuck B., Parker A., Sanderson J., Brown S.D.M., Scudamore C.L., Pusey C.D. (2019). Modification of an aggressive model of Alport Syndrome reveals early differences in disease pathogenesis due to genetic background. Sci. Rep..

[37] Takemon Y., Wright V., Davenport B., Gatti D.M., Sheehan S.M., Letson K., Savage H.S., Lennon R., Korstanje R. (2021). Uncovering Modifier Genes of X-Linked Alport Syndrome Using a Novel Multiparent Mouse Model. J. Am. Soc. Nephrol..

[38] Dufek B., Meehan D.T., Delimont D., Cheung L., Gratton M.A., Phillips G., Song W., Liu S., Cosgrove D. (2016). Endothelin A receptor activation on mesangial cells initiates Alport glomerular disease. Kidney Int..

[39] Kim J.J., David J.M., Wilbon S.S., Santos J.V., Patel D.M., Ahmad A., Mitrofanova A., Liu X., Mallela S.K., Ducasa G.M. (2021). Discoidin domain receptor 1 activation links extracellular matrix to podocyte lipotoxicity in Alport syndrome. EBioMedicine.

[40] Cosgrove D., Meehan D.T., Grunkemeyer J.A., Kornak J.M., Sayers R., Hunter W.J., Samuelson G.C. (1996). Collagen COL4A3 knockout: A mouse model for autosomal Alport syndrome. Genes Dev..

[41] Cosgrove D., Samuelson G., Meehan D.T., Miller C., McGee J., Walsh E.J., Siegel M. (1998). Ultrastructural, physiological, and molecular defects in the inner ear of a gene-knockout mouse model for autosomal Alport syndrome. Hear. Res..

[42] Miner J.H., Sanes J.R. (1996). Molecular and functional defects in kidneys of mice lacking collagen α3(IV): Implications for Alport syndrome. J. Cell Biol..

[43] Andrews K.L., Betsuyaku T., Rogers S.S., Michael Shipley J., Senior R.M., Miner J.H. (2000). Gelatinase B (MMP-9) is not essential in the normal kidney and does not influence progression of renal disease in a mouse model of alport syndrome. Am. J. Pathol..

[44] Korstanje R., Caputo C.R., Doty R.A., Cook S.A., Bronson R.T., Davisson M.T., Miner J.H. (2014). A mouse Col4a4 mutation causing Alport glomerulosclerosis with abnormal collagen α3α4α5(IV) trimers. Kidney Int..

[45] Arnold C.N., Xia Y., Lin P., Ross C., Schwander M., Smart N.G., Müller U., Beutler B. (2011). Rapid identification of a disease allele in mouse through whole genome sequencing and bulk segregation analysis. Genetics.

[46] Rheault M.N., Kren S.M., Thielen B.K., Mesa H.A., Crosson J.T., Thomas W., Sado Y., Kashtan C.E., Segal Y. (2004). Mouse model of X-linked Alport syndrome. J. Am. Soc. Nephrol..

[47] Gyoneva L., Segal Y., Dorfman K.D., Barocas V.H. (2013). Mechanical response of wild-type and Alport murine lens capsules during osmotic swelling. Exp. Eye Res..

[48] Hashikami K., Asahina M., Nozu K., Iijima K., Nagata M., Takeyama M. (2019). Establishment of X-linked Alport syndrome model mice with a Col4a5 R471X mutation. Biochem. Biophys. Rep..

[49] Bult C.J., Kadin J.A., Richardson J.E., Blake J.A., Eppig J.T. (2009). The mouse genome database: Enhancements and updates. Nucleic Acids Res..

[50] Naylor R.W., Morais M.R.P.T., Lennon R. (2021). Complexities of the glomerular basement membrane. Nat. Rev. Nephrol..

[51] Quinlan C., Rheault M.N. (2021). Genetic Basis of Type IV Collagen Disorders of the Kidney. Clin. J. Am. Soc. Nephrol..

[52] Suh J.H., Miner J.H. (2013). The glomerular basement membrane as a barrier to albumin. Nat. Rev. Nephrol..

[53] Wu Y., Ge G. (2019). Complexity of type IV collagens: From network assembly to function. Biol. Chem..

[54] Savige J., Storey H., Watson E., Hertz J.M., Deltas C., Renieri A., Mari F., Hilbert P., Plevova P., Byers P. (2021). Consensus statement on standards and guidelines for the molecular diagnostics of Alport syndrome: Refining the ACMG criteria. Eur. J. Hum. Genet..

[55] Vanacore R., Ham A.J.L., Voehler M., Sanders C.R., Conrads T.P., Veenstra T.D., Sharpless B., Dawson P.E., Hudson B.G. (2009). A Sulfilimine Bond Identified in Collagen IV. Science.

[56] Pedchenko V., Boudko S.P., Barber M., Mikhailova T., Saus J., Harmange J.-C., Hudson B.G. (2021). Collagen IVα345 dysfunction in glomerular basement membrane diseases. III. A functional framework for α345 hexamer assembly. J. Biol. Chem..

[57] Abrahamson D.R. (2012). Role of the Podocyte (and Glomerular Endothelium) in Building the GBM. Semin. Nephrol..

[58] St. John P.L., Abrahamson D.R. (2001). Glomerular endothelial cells and podocytes jointly synthesize laminin-1 and -11 chains. Kidney Int..

[59] Kashyan C.E., Kim Y., Lees G.E., Thorner P.S., Virtanen I., Miner J.H. (2001). Abnormal Glomerular Basement Membrane Laminins in Murine, Canine, and Human Alport Syndrome: Aberrant Laminin α2 Deposition Is Species Independent. J. Am. Soc. Nephrol..

[60] Abrahamson D.R., Prettyman A.C., Robert B., St. John P.L. (2003). Laminin-1 reexpression in Alport mouse glomerular basement membranes. Kidney Int..

[61] Zallocchi M., Johnson B.M., Meehan D.T., Delimont D., Cosgrove D. (2013). α1β1 Integrin/rac1-dependent mesangial invasion of glomerular capillaries in alport syndrome. Am. J. Pathol..

[62] Steenhard B.M., Vanacore R., Friedman D., Zelenchuk A., Stroganova L., Isom K., John S.P.L., Hudson B.G., Abrahamson D.R. (2012). Upregulated Expression of Integrin α1 in Mesangial Cells and Integrin α3 and Vimentin in Podocytes of Col4a3-Null (Alport) Mice. PLoS ONE.

[63] Cosgrove D., Meehan D.T., Delimont D., Pozzi A., Chen X., Rodgers K.D., Tempero R.M., Zallocchi M., Rao V.H. (2008). Integrin α1β1 regulates matrix metalloproteinases via p38 mitogen-activated protein kinase in mesangial cells: Implications for alport syndrome. Am. J. Pathol..

[64] Hahm K., Lukashev M.E., Luo Y., Yang W.J., Dolinski B.M., Weinreb P.H., Simon K.J., Li C.W., Leone D.R., Lobb R.R. (2007). αvβ6 Integrin Regulates Renal Fibrosis and Inflammation in Alport Mouse. Am. J. Pathol..

[65] Suleiman H., Zhang L., Roth R., Heuser J.E., Miner J.H., Shaw A.S., Dani A. (2013). Nanoscale protein architecture of the kidney glomerular basement membrane. eLife.

[66] Gross O., Girgert R., Beirowski B., Kretzler M., Kang H.G., Kruegel J., Miosge N., Busse A.C., Segerer S., Vogel W.F. (2010). Loss of collagen-receptor DDR1 delays renal fibrosis in hereditary type IV collagen disease. Matrix Biol..

[67] Richter H., Satz A.L., Bedoucha M., Buettelmann B., Petersen A.C., Harmeier A., Hermosilla R., Hochstrasser R., Burger D., Gsell B. (2018). DNA-Encoded Library-Derived DDR1 Inhibitor Prevents Fibrosis and Renal Function Loss in a Genetic Mouse Model of Alport Syndrome. ACS Chem. Biol..

[68] Rubel D., Frese J., Martin M., Leibnitz A., Girgert R., Miosge N., Eckes B., Müller G.A., Gross O. (2014). Collagen receptors integrin alpha2beta1 and discoidin domain receptor 1 regulate maturation of the glomerular basement membrane and loss of integrin alpha2beta1 delays kidney fibrosis in COL4A3 knockout mice. Matrix Biol..

[69] Sannomiya Y., Kaseda S., Kamura M., Yamamoto H., Yamada H., Inamoto M., Kuwazuru J., Niino S., Shuto T., Suico M.A. (2021). The role of discoidin domain receptor 2 in the renal dysfunction of alport syndrome mouse model. Ren. Fail..

[70] Rao V.H., Meehan D.T., Delimont D., Nakajima M., Wada T., Gratton M.A., Cosgrove D. (2006). Role for macrophage metalloelastase in glomerular basement membrane damage associated with Alport syndrome. Am. J. Pathol..

[71] Delimont D., Dufek B.M., Meehan D.T., Zallocchi M., Gratton M.A., Phillips G., Cosgrove D. (2014). Laminin α2-mediated focal adhesion kinase activation triggers Alport glomerular pathogenesis. PLoS ONE.

[72] Abrahamson D.R., Isom K., Roach E., Stroganova L., Zelenchuk A., Miner J.H., St. John P.L. (2007). Laminin compensation in collagen α3(IV) knockout (Alport) glomeruli contributes to permeability defects. J. Am. Soc. Nephrol..

[73] Ding F., Wickman L., Wang S.Q., Zhang Y., Wang F., Afshinnia F., Hodgin J., Ding J., Wiggins R.C. (2017). Accelerated podocyte detachment and progressive podocyte loss from glomeruli with age in Alport Syndrome. Kidney Int..

[74] Clauss S., Gross O., Kulkarni O., Avila-Ferrufino A., Radomska E., Segerer S., Eulberg D., Klussmann S., Anders H.J. (2009). Ccl2/Mcp-I blockade reduces glomerular and interstitial macrophages but does not ameliorate renal pathology in co//agen4A3-deficient mice with autosomal recessive alport nephropathy. J. Pathol..

[75] Gast C., Pengelly R.J., Lyon M., Bunyan D.J., Seaby E.G., Graham N., Venkat-Raman G., Ennis S. (2016). Collagen (COL4A) mutations are the most frequent mutations underlying adult focal segmental glomerulosclerosis. Nephrol. Dial. Transplant..

[76] Ishiko S., Tanaka A., Takeda A., Hara M., Hamano N., Koizumi M., Ueno T., Hayashi H., Kondo A., Nagai S. (2021). Utility of glomerular Gd-IgA1 staining for indistinguishable cases of IgA nephropathy or Alport syndrome. Clin. Exp. Nephrol..

[77] Morinière V., Dahan K., Hilbert P., Lison M., Lebbah S., Topa A., Bole-Feysot C., Pruvost S., Nitschke P., Plaisier E. (2014). Improving mutation screening in familial hematuric nephropathies through next generation sequencing. J. Am. Soc. Nephrol..

[78] Li B., Zhang Y., Wang F., Nair V., Ding F., Xiao H., Yao Y., Kretzler M., Ju W., Ding J. (2018). Urinary epidermal growth factor as a prognostic marker for the progression of Alport syndrome in children. Pediatr. Nephrol..

[79] Temme J., Kramer A., Jager K.J., Lange K., Peters F., Müller G.A., Kramar R., Heaf J.G., Finne P., Palsson R. (2012). Outcomes of male patients with Alport syndrome undergoing renal replacement therapy. Clin. J. Am. Soc. Nephrol..

[80] Savva I., Pierides A., Deltas C. (2016). RAAS inhibition and the course of Alport syndrome. Pharmacol. Res..

[81] Zhang F., Liu H., Liu D., Liu Y., Li H., Tan X., Liu F., Peng Y., Zhang H. (2017). Effects of RAAS Inhibitors in Patients with Kidney Disease. Curr. Hypertens. Rep..

[82] Yamamura T., Horinouchi T., Nagano C., Omori T., Sakakibara N., Aoto Y., Ishiko S., Nakanishi K., Shima Y., Nagase H. (2020). Genotype-phenotype correlations influence the response to angiotensin-targeting drugs in Japanese patients with male X-linked Alport syndrome. Kidney Int..

[83] Temme J., Peters F., Lange K., Pirson Y., Heidet L., Torra R., Grunfeld J.P., Weber M., Licht C., Müller G.A. (2012). Incidence of renal failure and nephroprotection by RAAS inhibition in heterozygous carriers of X-chromosomal and autosomal recessive Alport mutations. Kidney Int..

[84] Zhang Y., Böckhaus J., Wang F., Wang S., Rubel D., Gross O., Ding J. (2021). Genotype–phenotype correlations and nephroprotective effects of RAAS inhibition in patients with autosomal recessive Alport syndrome. Pediatr. Nephrol..

[85] Gross O., Licht C., Anders H.J., Hoppe B., Beck B., Tönshoff B., Höcker B., Wygoda S., Ehrich J.H.H., Pape L. (2012). Early angiotensin-converting enzyme inhibition in Alport syndrome delays renal failure and improves life expectancy. Kidney Int..

[86] Jarad G., Knutsen R.H., Mecham R.P., Miner J.H. (2016). Albumin contributes to kidney disease progression in alport syndrome. Am. J. Physiol. Ren. Physiol..

[87] Gross O., Tönshoff B., Weber L.T., Pape L., Latta K., Fehrenbach H., Lange-Sperandio B., Zappel H., Hoyer P., Staude H. (2020). A multicenter, randomized, placebo-controlled, double-blind phase 3 trial with open-arm comparison indicates safety and efficacy of nephroprotective therapy with ramipril in children with Alport’s syndrome. Kidney Int..

[88] Gross O., Beirowski B., Koepke M.L., Kuck J., Reiner M., Addicks K., Smyth N., Schulze-Lohoff E., Weber M. (2003). Preemptive ramipril therapy delays renal failure and reduces renal fibrosis in COL4A3-knockout mice with Alport syndrome. Kidney Int..

[89] Kashtan C.E., Gross O. (2021). Clinical practice recommendations for the diagnosis and management of Alport syndrome in children, adolescents, and young adults–an update for 2020. Pediatr. Nephrol..

[90] MN R., WE S. (2020). Long-term ACE inhibition in Alport syndrome: Are the benefits worth the risks?. Kidney Int..

[91] Stenvinkel P., Chertow G.M., Devarajan P., Levin A., Andreoli S.P., Bangalore S., Warady B.A. (2021). Chronic Inflammation in Chronic Kidney Disease Progression: Role of Nrf2. Kidney Int. Rep..

[92] Rubel D., Stock J., Ciner A., Hiller H., Girgert R., Müller G.A., Gross O. (2014). Antifibrotic, nephroprotective effects of paricalcitol versus calcitriol on top of ACE-inhibitor therapy in the COL4A3 knockout mouse model for progressive renal fibrosis. Nephrol. Dial. Transplant..

[93] Gomez I.G., MacKenna D.A., Johnson B.G., Kaimal V., Roach A.M., Ren S., Nakagawa N., Xin C., Newitt R., Pandya S. (2015). Anti-microRNA-21 oligonucleotides prevent Alport nephropathy progression by stimulating metabolic pathways. J. Clin. Investig..

[94] De Zeeuw D., Coll B., Andress D., Brennan J.J., Tang H., Houser M., Correa-Rotter R., Kohan D., Heerspink H.J.L., Makino H. (2014). The endothelin antagonist atrasentan lowers residual albuminuria in patients with type 2 diabetic nephropathy. J. Am. Soc. Nephrol..

[95] Heerspink H.J.L., Parving H.H., Andress D.L., Bakris G., Correa-Rotter R., Hou F.F., Kitzman D.W., Kohan D., Makino H., McMurray J.J.V. (2019). Atrasentan and renal events in patients with type 2 diabetes and chronic kidney disease (SONAR): A double-blind, randomised, placebo-controlled trial. Lancet.

[96] Rubel D., Zhang Y., Sowa N., Girgert R., Gross O. (2021). Organoprotective Effects of Spironolactone on Top of Ramipril Therapy in a Mouse Model for Alport Syndrome. J. Clin. Med..

[97] Juurlink D.N., Mamdani M.M., Lee D.S., Kopp A., Austin P.C., Laupacis A., Redelmeier D.A. (2004). Rates of Hyperkalemia after Publication of the Randomized Aldactone Evaluation Study. N. Engl. J. Med..

[98] Koepke M.L., Weber M., Schulze-Lohoff E., Beirowski B., Segerer S., Gross O. (2007). Nephroprotective effect of the HMG-CoA-reductase inhibitor cerivastatin in a mouse model of progressive renal fibrosis in Alport syndrome. Nephrol. Dial. Transplant..

[99] Trachtman H., Nelson P., Adler S., Campbell K.N., Chaudhuri A., Derebail V.K., Gambaro G., Gesualdo L., Gipson D.S., Hogan J. (2018). DUET: A Phase 2 Study Evaluating the Efficacy and Safety of Sparsentan in Patients with FSGS. J. Am. Soc. Nephrol..

[100] Komers R., Diva U., Inrig J.K., Loewen A., Trachtman H., Rote W.E. (2020). Study Design of the Phase 3 Sparsentan Versus Irbesartan (DUPLEX) Study in Patients With Focal Segmental Glomerulosclerosis. Kidney Int. Rep..

[101] Weinstock B.A., Feldman D.L., Fornoni A., Gross O., Kashtan C.E., Lagas S., Lennon R., Miner J.H., Rheault M.N., Simon J.F. (2020). Clinical trial recommendations for potential Alport syndrome therapies. Kidney Int..

[102] Omachi K., Kaseda S., Yokota T., Kamura M., Teramoto K., Kuwazuru J., Kojima H., Nohara H., Koyama K., Ohtsuki S. (2021). Metformin ameliorates the severity of experimental Alport syndrome. Sci. Rep..

[103] Suh S.H., Choi H.S., Kim C.S., Kim I.J., Ma S.K., Scholey J.W., Kim S.W., Bae E.H. (2019). Olmesartan attenuates kidney fibrosis in a murine model of alport syndrome by suppressing tubular expression of TGFβ. Int. J. Mol. Sci..

[104] Suh S.H., Mathew A.P., Choi H.S., Vasukutty A., Kim C.S., Kim I.J., Ma S.K., Kim S.W., Park I.-K., Bae E.H. (2021). Kidney-accumulating olmesartan-loaded nanomicelles ameliorate the organ damage in a murine model of Alport syndrome. Int. J. Pharm..

